# Impact of Macrodiols on the Morphological Behavior of H_12_MDI/HDO-Based Polyurethane Elastomer

**DOI:** 10.3390/polym13132060

**Published:** 2021-06-23

**Authors:** Shazia Naheed, Mohammad Zuber, Mahwish Salman, Nasir Rasool, Zumaira Siddique, Mohammed Rafi Shaik, Mohammed A. F. Sharaf, Abdelatty Abdelgawad, Doumbia Sekou, Emad Mahrous Awwad

**Affiliations:** 1 Department of Chemistry, Government College University, Faisalabad 38030, Pakistan; nasirrasool@gcuf.edu.pk (N.R.); zumairasiddique@gmail.com (Z.S.); 2 Department of Chemistry, University of Lahore, Lahore 54000, Pakistan; muhammad.zuber@chem.uol.edu.pk; 3 Department of Biochemistry, Government College University, Faisalabad 38030, Pakistan; mahwish.gene@gmail.com; 4 Department of Chemistry, College of Science, King Saud University, P.O. Box 2455, Riyadh 11451, Saudi Arabia; 5 Department of Industrial Engineering, College of Engineering, King Saud University, P.O. Box 800, Riyadh 11421, Saudi Arabia; mfsharaf@ksu.edu.sa (M.A.F.S.); aesayed@ksu.edu.sa (A.A.); 6 Department of Agricultural Extension and Rural Society, College of Food and Agriculture Sciences, King Saud University, P.O. Box 2460, Riyadh 11451, Saudi Arabia; 442106474@student.ksu.edu.sa; 7 Department of Electrical Engineering, College of Engineering, King Saud University, P.O. Box 800, Riyadh 11421, Saudi Arabia; 436107822@student.ksu.edu.sa

**Keywords:** polycaprolactone diol, polyurethane, 4,4′-cyclohexamethylene diisocyanate, 1,6-hexanediol, atomic force microscopy

## Abstract

In this study, we evaluated the morphological behavior of polyurethane elastomers (PUEs) by modifying the soft segment chain length. This was achieved by increasing the soft segment molecular weight (*Mn* = 400–4000 gmol^−1^). In this regard, polycaprolactone diol (PCL) was selected as the soft segment, and 4,4′-cyclohexamethylene diisocyanate (H_12_MDI) and 1,6-hexanediol (HDO) were chosen as the hard segments. The films were prepared by curing polymer on Teflon surfaces. Fourier transform infrared spectroscopy (FTIR) was utilized for functional group identification in the prepared elastomers. FTIR peaks indicated the disappearance of −NCO and −OH groups and the formation of urethane (NHCOO) groups. The morphological behavior of the synthesized polymer samples was also elucidated using scanning electron microscopy (SEM) and atomic force microscopy (AFM) techniques. The AFM and SEM results indicated that the extent of microphase separation was enhanced by an increase in the molecular weight of PCL. The phase separation and degree of crystallinity of the soft and hard segments were described using X-ray diffraction (XRD). It was observed that the degree of crystallinity of the synthesized polymers increased with an increase in the soft segment’s chain length. To evaluate hydrophilicity/hydrophobicity, the contact angle was measured. A gradual increase in the contact angle with distilled water and diiodomethane (38.6°–54.9°) test liquids was observed. Moreover, the decrease in surface energy (46.95–24.45 mN/m) was also found to be inconsistent by increasing the molecular weight of polyols.

## 1. Introduction

The importance of PU elastomers is increasing because of their broad range of properties and increasing number of applications [1,2,3]. The macromolecules of PUEs are composed of relatively long, flexible segments (SSs) and short, rigid segments (HSs), with alternating polydisperse blocks. As a result of the phase separation process, a soft phase is formed from the SSs, and a hard phase is formed from the HSs. The soft phases, with minor glass transition temperature (Tg) values, usually consist of polyethers, polyesters or polyols, whose molecular weight range is from 400 to 5000. In contrast, the diisocyanate and chain extenders form the polar rigid hard segments, possessing high Tg of the hard phase. [2,4,5,6,7].

Polyurethane elastomers demonstrated microphase separation due to thermodynamic immiscibility between the polyol (soft segment) and urethane (hard segment). This double-phase microdomain structure shown by PUEs provides them with their better mechanical properties [2,4,8,9].

Importantly, the phase separation of the microdomain structure is not only influenced by the diisocyanate structure, molecular weight, cross-linking density, and ratio of the macrodiol and the chain extender [4,5,10], but the thermal stability of the material is also an influencing factor [11]. Moreover, the reaction conditions, such as temperature, pH and pressure, can also significantly change the domain structure [12]. Therefore, the dynamic and static properties of PUEs can be modified by selecting different monomers, altering the cross-linking process of the polyfunctional compound or by simply changing the reaction conditions [13].

To elucidate the relationships between morphology, chemical structural design, and the chemical properties of polymers, different physiochemical techniques were utilized [14]. It was known that macroscopic properties are dependent on the structure, size, and shape of the soft and hard phase [10]. In addition, it was noted that both the surface properties were also improved by changing the polyol’s molecular weight, with microphase separation playing a role in thermodynamics, hard-phase packing in the hard domain and soft-phase crystallinity [15].

The present study enforces the morphological analyses of PUEs. The morphological effect on the material’s behavior was evaluated by characterizing the prepared material. As PUEs are formed by combining hard (isocyanate) and elastic (polyol) parts, modification of these parts gives the polyurethane elastomers novel qualities. The PUEs were synthesized by using a wide range of molecular weights for the PCL polyols (*Mn =* 400, 750, 1000, 2000, 3000 and 4000 gmol^−1^), which were utilized as soft segments (SSs). The H_12_MDI and HDO were used as ingredients to develop urethane linkage as hard segments (HSs) using the pre-polymer method. The reaction chemistry, influence of structure on morphological behavior, process ability and reactivity of the final product were studied. To the best of our knowledge, there are no other reports about this kind of polymer. Moreover, efforts have been made to elucidate the surface properties, crystallinity and hydrophilicity of polymeric materials through FTIR, SEM, AFM, XRD, contact angle and surface energy technologies. Meanwhile, our published article discusses the consequential effect of increasing the molecular weight of PCL on thermomechanical properties, assessed using TGA, DSC and DMTA; molecular characterization, assessed using FTIR; and the shape memory behavior of these polymers [16].

## 2. Methodology

### 2.1. Materials and Synthesis

Analytical-grade chemicals, including 4,4′-dicyclohexylemethane diisocyanate (H_12_MDI) (2 mol), 1,6-hexanediol (HDO) (1.2 mol) and polycaprolactone diols (ranges *Mn* 400–4000 (0.8 mol)), were used to synthesize polyurethanes elastomers using step-growth polymerization (Table 1). The detailed methodology for the preparation of PUEs has been reported in our earlier published work [16].

The PUEs were stirred in a four-necked round-bottom flask, and the polycaprolactone diol was added and stirred for a few minutes at 60–70 °C. H_12_MDI was poured into the reaction chamber, and then the temperature was increased to 90–100 °C. The stirring continued for 2 h until the pre-polymer was synthesized [16]. Finally, 1,6-HDO was introduced into the pre-polymer reaction mixture as the chain extender (Table 1). The reaction was continued for a further 30 min at 90–100 °C. The appearance of a viscous and transparent material in the reaction chamber signaled the successful synthesis of PUE. Titration with *n*-butylamine (ASTM D 2572-80) was conducted to obtain the NCO contents of the polymer. The synthesis of the pre-polymer was confirmed using FTIR spectroscopy.

**Figure 1:**
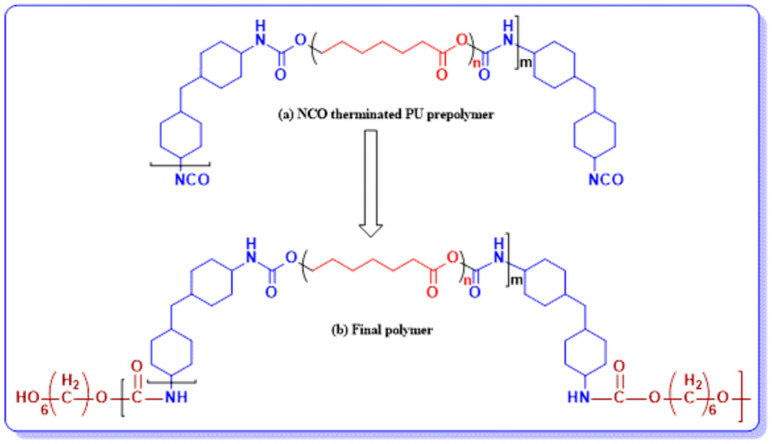
Polyurethane elastomer synthesis using polycaprolactone diol as SS: (**a**) pre-polymer and (**b**) final polyurethane.

By changing the molecular weight of the polycaprolactone diols in the PU pre-polymer, eight samples were synthesized by following the above-mentioned procedure. Figure 1 displays the schematic elucidation of the route adopted for the preparation of the polymer.

### 2.2. Characterization

The Equinox 55 Fourier Transform Infrared (FTIR) spectrometer (Bruker, Germany) produced FTIR spectra of the prepared PUEs with a range of 750–4000 cm^−1^, generated using attenuated total reflection (ATR) assembly. X-ray diffractograms of the PUEs were attained in dispersion range (2θ) of 5–70° by a Siemens D-5000 diffractometer with Cu-Ka radiations (λ = 1.54059 nm, 40 KV and 40 mA) at 25 °C. Two test liquids (double-distilled water and diiodomethane) were used to calculate the contact angle with the Krüss G10 contact angle measuring system. Surface tension was measured using Wu’s method. The surface topography of the fractured side was studied using scanning electron microscopy (SEM) at 2 kx, 5 kx and 15 kx after it was covered with a thin, gold layer using AFM (Germany) in tapping mode.

## 3. Results and Discussion

### 3.1. Molecular Characterization

**Figure 2:**
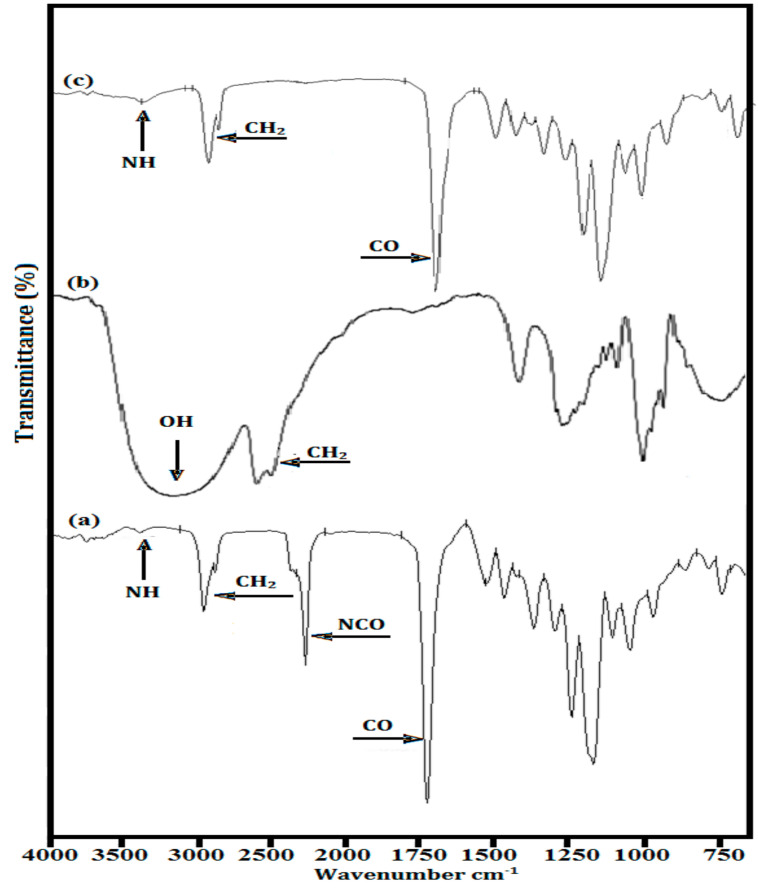
FTIR spectra of (**a**) pre-polymer, (**b**) HDO and (**c**) PU8.

FTIR spectra of the isocyanate, −NCO-terminated pre-polymer, 1,6-hexanediol (HDO) chain extender and PU are presented to explain the structural characterization (Figure 2). The peaks of PUEs for urethane groups were identified at 3323–3328 cm^−1^ for −NH− stretching, as reported in [17], the urethane carbonyl bond appeared at 1652–1725 cm^−1^, and the peaks at 2851–2862 and 2921–2935 cm^−1^ were attributed [16] to –CH_2_– groups (symmetric and asymmetric stretching vibrations), respectively [18]. The other observed peaks for all synthesized polyurethanes appeared at 1520–1524 cm^−1^ (–NH– deformations), 1442–1465 cm^−1^ (–CH_2_– bending vibration), 1412–1431 cm^−1^ (–CH– bending vibration) and 1306–1362 cm^−1^ (–CH_2_– wagging), which verified the new synthesized products as having formed –NHCOO– groups. Using the above-discussed peak values, it is clear that all polyurethanes were successfully synthesized; the appearance of –NH– peaks showed complete consumption of –NCO-terminated pre-polymers. It was noted that the MW of polycaprolactone diol and the peak intensity of –NH– stretching vibration showed reciprocal trends, and the sharpness of the urethane carbonyl peaks was observed to continuously increase as the molecular weight of the SS increased. In PU with high molecular weight PCL, the number of ester linkages gradually increased without any change in –NH groups. As such, the relative peak intensity of the −NH group decreased as compared with that of the –C=O group. In addition, by increasing the length of the polycaprolactone diol, the relative amount of –NH groups also decreased per unit weight of PU.

### 3.2. X-Ray Diffraction Studies (XRD)

**Figure 3:**
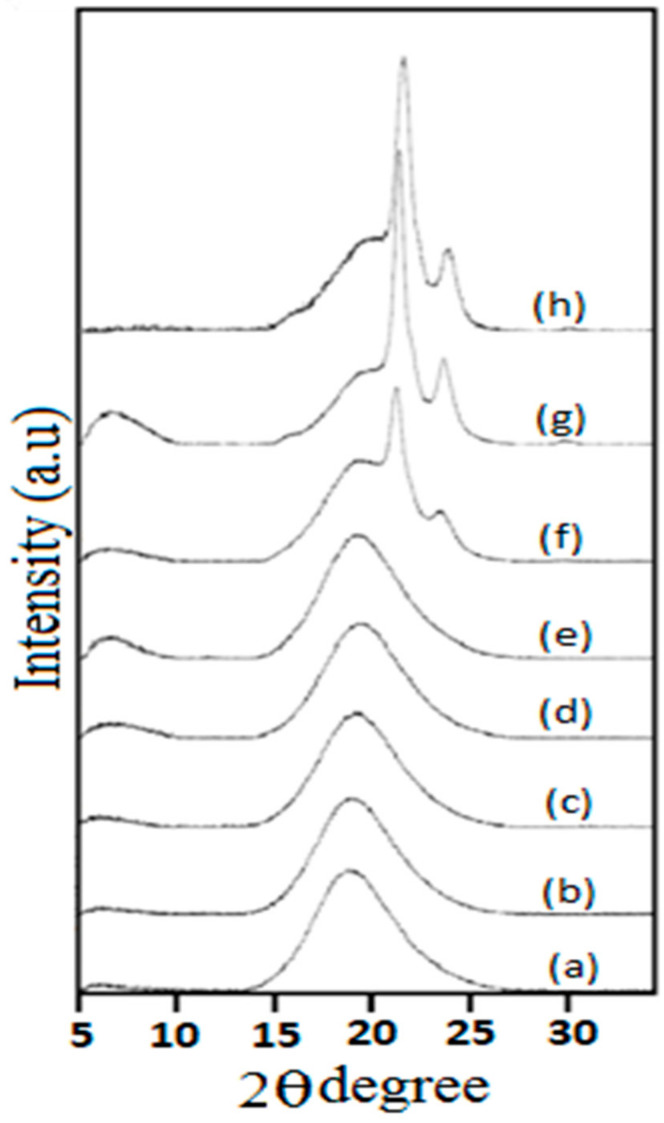
X-ray diffractograms of PUEs sorted by varying molecular weight of PCLs (ascending order).

To determine the dependence of the crystalline structure on the molecular weight of polyols, XRD analysis was used. Phase separation and the degree of crystallinity of the SS and HS were evaluated using XRD. In segmented polyurethanes, the phase separation is dependent upon the thermodynamic incompatibility and structural regularity of soft segments. XRD investigations showed that in the polyurethane backbone, the number of methylene (–CH_2_) units affected the crystallinity of the polymer, and this increased with the increasing PCL chain length (Figure 3). It was observed that localized peak intensity at 2θ = 20° generated better-defined peaks when the molecular weight of the polyol was increased. It was observed that PUE7 and PUE8 with *Mn* 3000 and 4000, respectively, had sharper and higher peak intensities, which resulted in a greater degree of chain orientation. This increase in length may be enhanced in phase segregation and may be a result of SS mobility. The broader and lower peak height of PUE1 indicated the poor crystalline structure of the polymer sample [19]. The degree of crystallinity (*X_C_)* was found by using the following Equation:

(1) $$X_{C}=\frac{A_{C}}{A_{T}}*100\%$$

where the area of crystalline peaks is *A_C_*, and the total area of amorphous and crystalline peaks is *A_T._*

The soft phase SS showed improved crystallinity due to the stable disposition of their molecular structures. Notably, it was observed that when hard segments were present at high concentrations, the diffraction peaks observed were in the range of 2θ = 11.12°. Consequently, the degree of crystallinity of these polymers (Table 2) was mainly due to the soft segments, which provided more freedom to hard segmental arrangements and enhanced the orientation of the crystallization [20,21].

### 3.3. Studies of Surface Morphology

#### 3.3.1. Measurements of Contact Angle

The measurement of contact angles enabled us to calculate hydrophilicity/hydrophobicity by using diiodomethane and double-distilled water. The precise measurement of the contact angles was performed between the surface of PU films and test liquids. When water was the test liquid, the contact angle values changed from 86° to 108° as the molecular weight of polyol increased (Table 3). When diiodomethane was the test liquid, the contact angle values increased from 38.6° to 54.9°. This showed that hydrophobicity increased as the chain length of the soft segments of the synthesized polymer increased. This may be due to the increase in chain length and the conformational orientation of the SSs, which enhances the possibility of packing of hard segments (HSs). Hence, these crystalline HSs embedded and randomly dispersed in soft segment (SSs) carrying polar ester groups. These ester groups on the surface interact with the solvent and help to increase the wettability of the surface. Furthermore, the increased chain packing of SSs internally satisfies the intermolecular polymer interaction. However, it makes the surface more hydrophilic [22].

#### 3.3.2. Surface Free Energy

Surface tension with polar and dispersive portions was calculated using Wu’s method [23]. A decrease in surface energy was seen as the molecular weight of the polyol increased (Table 3). This occurred due to an increase in the contact angle. The contact angle of a liquid with a solid surface can be associated with solid surface energy (^γ^_S_), liquid surface tension (^γ^_L_), and solid–liquid interfacial tension (^γ^_SL_) using Young^’^s Equation:

cos θ = ^γ^_S_ − ^γ^_SL/_^γ^_L_ (2)

The results in Table 4 show that the contact angle would increase if there was lower solid surface energy or higher liquid surface tension. As such, a solid surface can be made more wettable either by lowering the surface energy of solids or by increasing the surface tension of liquids [24]. Thus, it was found that increasing the length of soft segment results in a decrease in surface energy (i.e., an increase in chain mobility in the synthesized polymer). It was also observed that the dispersive part increased with increased polyol chain length.

### 3.4. Morphology

#### 3.4.1. Scanning Electron Microscopy (SEM)

**Figure 4:**
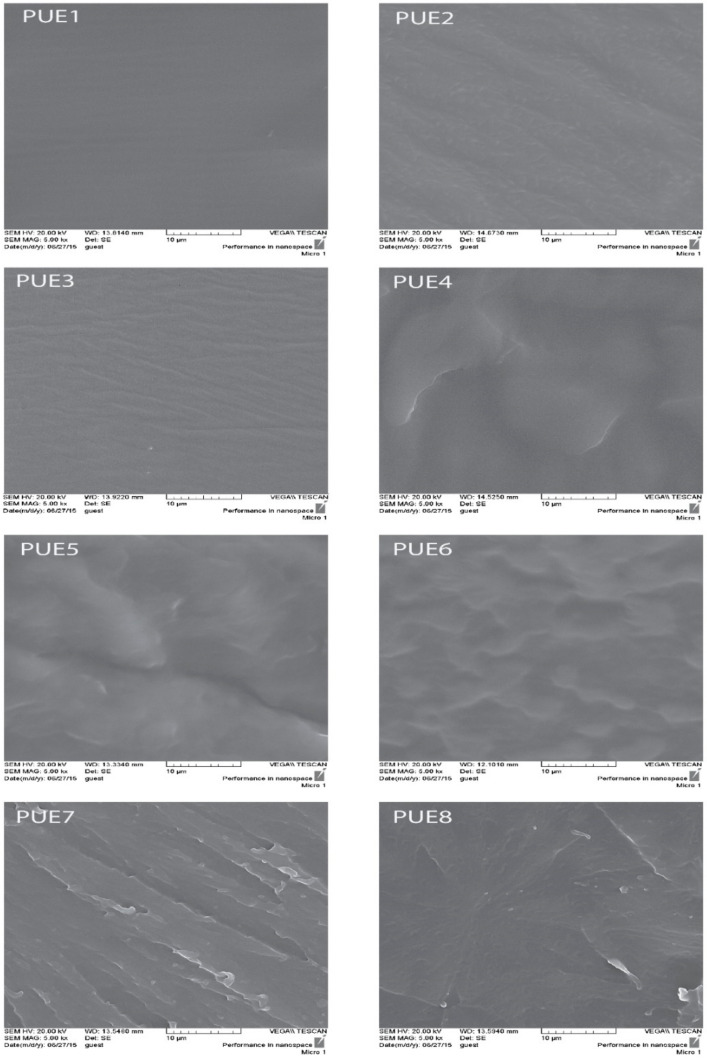
Scanning electron images (SEM) of polyurethane elastomers.

The morphology of the matrix system was examined using SEM (Figure 4). Microphase separation occurs due to thermodynamic incompatibility among hard and soft segments of segmented polyurethanes [22,25]. Moreover, chemical composition, hydrogen bonding, sequence length of the hard segment etc. may also influence phase separation. In polyurethane, phase separation is considered to be closely related to hydrogen bonding [26]. PUE1–PUE3 polyurethanes, made of PCL with a lower MW [16,27], have a structure different from that of polyurethanes made using PCL with MW > 1250. With more than 55% of its content being flexible segments, the polyurethane matrix is a hard phase in which the flexible segments or their agglomerates are dispersed. When polyurethanes contain up to about 55% flexible segments, the polyurethane matrix is the soft phase, and the rigid segments or their agglomerates are dispersed in it. This is probably similar in the tested polyurethanes. For PUE1–PUE3, the matrix of polyurethanes is the hard phase, and for the remaining PUE4–PUE8 materials, the matrix is the soft phase. Therefore, the microstructure of PUE1–PUE3 is different from that of PUE4–PUE8, which is noticeable at higher magnification of images. The images of the breakthroughs of the PUE1–PUE3 samples resemble microfibrils on the surfaces with visible ovals of the soft-phase agglomerates, which is well illustrated by the breakthrough of the PUE2 sample. In the case of PUE4 and PUE5 samples, the soft-phase matrix is in amorphous form. The remaining samples are in the crystalline phase (PUE6, PUE7 and PUE8), which significantly changes the surface morphology of the brittle fractures of these samples. The surfaces of the PUE4 and PUE5 samples show agglomerates of hard domains in the form of ovals located in the soft phase. With the change in the proportion of the crystal phase in PUE6, PUE7 and PUE8, the nature of the fracture structure of these materials changes. In the case of PUE6, tearing out of the crystallite ovals from the matrix made of the soft phase is visible. In the area of these tears, clusters of agglomerates of hard domains are visible. On the surface of the fracture of the PUE7 sample, strand-like cuts are visible, which are probably agglomerates of crystallographic plates made of ordered segments of the soft phase of PCL. Ovals of agglomerates of hard domains are visible on the surfaces between the tears and inside them. In the case of the PUE8 fracture, the structure of the spherulite is visible, on the surface of which the ovals of the hard domains are visible. The spherulite visible in the image is about 30 µm. On that basis, PUE8 was studied using AFM to visualize the microstructure.

#### 3.4.2. Atomic Force Microscopy (AFM)

**Figure 5:**
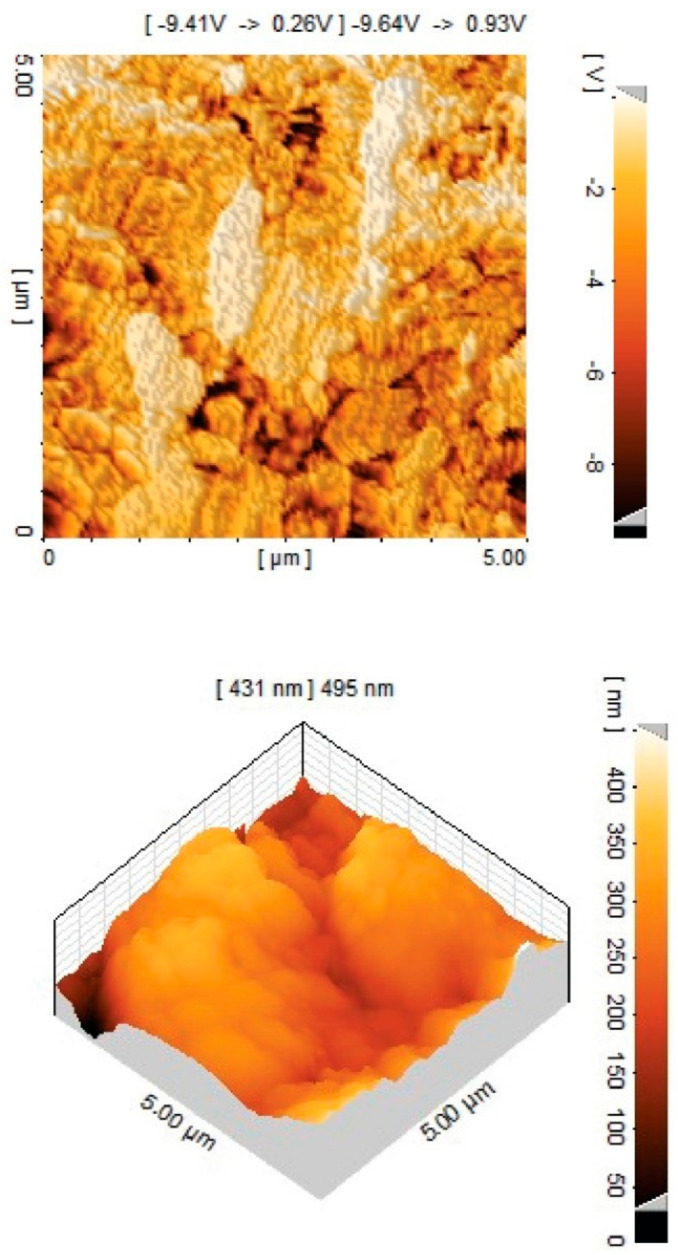
Three-dimensional AFM image of polyurethane sample (PUE8).

Atomic force microscopy (AFM) was utilized for the visualization of surface microstructure and polymer flexibility. Figure 5 shows images of soft and hard domains. In addition, the physiochemical nature (polarity and wettability), disparity shape orientation, polymer domain size, and surface uniformity in space were distinctly extracted from AFM data. This technique was also used for the determination of phase microstructure in representing PUE8. AFM is also an important tool for interpreting the structural morphology at the nano-scale level. Tapping mode AFM was used to image the morphology of the synthesized samples possessing different proportions of soft and hard segments. The incompatibility of both segments caused phase separation, which was influenced by segmental length, hydrogen bonding and crystallization level [28].

The present study showed the morphology of PUE8 films. which can be seen in Figure 5. The darker regions correspond to soft segments (PCL), and lighter regions correspond to hard-ordered domains or crystalline regions in polyurethanes [29]. The oval structures visible in the 3D image (lower AFM image) are probably agglomerates of hard domains, as their size is about 2–3 µm, while the PUE 8 spherulites in the SEM image are much larger.

Moreover, in regard to polyurethane composition, it was also reported [30] that hydrogen bonding was present in urethane groups, which may be the cause of phase segregation. The higher molecular weight of PCL (4000 gmol^−1^) provided ordered and compact arrangements of the soft and hard phases. However, various factors can be responsible for phase segregation in PU, such as the dimensions of SS and HS, the polarity of groups, the chemical nature of PU and molecular weight [31]. Furthermore, the absence of globules and the presence of short and irregular hard segments on the entire surface were observed, which may provide structural reinforcement due to physical crosslink [32].

## 4. Conclusions

Various samples of polyurethane elastomers (PUEs) with low-to-high molecular weights of PCL (macrodiol), H_12_MDI (diisocyanate) and HDO chain extenders were synthesized and analyzed using FTIR. The appearance of −NH peaks along with the disappearance of –NCO and –OH aligned with the proposed polyurethane structures. Morphological analysis is an important tool for correlating the structure–property relationship of PUEs. SEM and AFM techniques provided topographical images of the samples, with the low molecular weight PCL samples displaying a clear, smooth and homogeneous microstructure without any deformation. In addition, clear phase segregation was observed in the high molecular weight PCL samples. This significant change in phase segregation is due to the soft and hard domains. Micrographs demonstrated that phase segregation depended upon the increasing molecular weight of the macrodiol. It was observed that the high molecular weight of PCL helped with the order and free arrangement of soft and hard segments. XRD was used to study the crystalline behavior of polyurethane films and demonstrated increasing crystallinity with successive increases in the macrodiol chain length. Furthermore, the reciprocal trend of contact angle increasing as surface energy decreased exhibited the active relationship between hydrophilicity and flexible segment length. As such, the investigation of the morphological behavior of synthesized elastomers explained the dependence of phase segregation, crystallinity and hydrophilicity on the molecular weight of polyol in the PUE backbone.

## Figures and Tables

**Table 1:** Sample codes, PCL molecular weight and the molar ratio of reactants in a series of molecular weight-based PUE samples.

Sr. No.	SamplesCode	PCL (MW)	Molar Ratio of (H_12_MDI/PCL/HDO)	Percentage of Soft Segments (%) SS
1	PUE1	400	2:0.8:1.2	34.86
2	PUE2	750	2:0.8:1.2	50.08
3	PUE3	1000	2:0.8:1.2	57.22
4	PUE4	1250	2:0.8:1.2	62.58
5	PUE5	1600	2:0.8:1.2	68.16
6	PUE6	2000	2:0.8:1.2	72.79
7	PUE7	3000	2:0.8:1.2	80.05
8	PUE8	4000	2:0.8:1.2	84.25

**Table 2:** Degree of crystallinity calculated from the XRD pattern.

Samples	Degree of Crystallinity (%)
PUE1	-
PUE2	-
PUE3	-
PUE4	-
PUE5	-
PUE6	17.2
PUE7	22.5
PUE8	33.4

**Table 3:** Contact angle’s variation with DDW and DIM, sorted by increasing molecular weight of PCL.

Sr. No.	Sample Code	Contact Angle (θ) with Water and Diiodomethane			
		DDW		DIM	
1	PUE 1	86.0	86.0	38.6	38.6
2	PUE 2	86.9	86.9	44.6	44.6
3	PUE 3	89.2	89.2	46.8	46.8
4	PUE 4	91.4	91.4	48.1	48.1
5	PUE 5	93.2	93.2	49.7	49.7
6	PUE 6	94.2	94.2	50.5	50.5
7	PUE 7	98.9	98.9	52	52
8	PUE 8	108	108	54.9	54.9

DDW = double-distilled water, DIM = diiodomethane.

**Table 4:** Data of the total surface energy calculated using Wu’s method.

Sr. No.	Sample Code	Surface Free Energy (Wu’s Method)		
		Polar Portion (mN/m)	Disperse Portion (mN/m)	Total Surface Energy (mN/m)
1	PUE 1	2.53	44.42	46.95
2	PUE 2	1.69	43.32	45.01
3	PUE 3	1.42	36.92	38.34
4	PUE 4	1.27	34.05	35.32
5	PUE 5	0.37	33.88	34.25
6	PUE 6	1.60	30.39	31.99
7	PUE 7	1.13	29.53	30.66
8	PUE 8	3.96	20.49	24.45

## Data Availability

Data are contained within the article.
